# Social inequalities and the environmental crisis: need for an intergenerational alliance: Tenth anniversary of Frontiers in Public Health

**DOI:** 10.3389/fpubh.2023.1226961

**Published:** 2023-08-30

**Authors:** Paolo Vineis, Ajay Gambhir

**Affiliations:** ^1^MRC Centre for Environment and Health, School of Public Health, Imperial College London, London, United Kingdom; ^2^Grantham Institute for Climate Change and the Environment, Imperial College London, South Kensington Campus, London, United Kingdom

**Keywords:** environment, social inequalities, Civil Economics, inter-generational equity, Green Deal

## Social inequalities and ecological crisis have the same historical roots

Thomas Piketty (1) has described the close relationship between the development of the “extractive” economy that led to the current environmental crisis, and the origins of social inequalities in the world, between countries and within countries. Both problems date back to the era of colonialism, when massive amounts of natural and human resources were literally extracted from those that became low-income countries. To this extraction process science was not extraneous. Let us consider the immense scientific efforts, particularly classificatory, carried out by the explorers of the 18th and 19th centuries, who sent to Kew Gardens in London and to the natural science museums in Berlin, Paris, London, Washington and New York the specimens they gradually discovered. Scientists such as von Humboldt, Wallace and Darwin were deeply involved in this work of discovery, extraction and classification. Von Humboldt, for example, explored Central and South America in the late 1700s, classified 60,000 plants and described entirely new natural phenomena. At the end of his life, in Berlin, he wrote a most ambitious work, “The Cosmos,” which denotes the universalistic aims of these great explorers and naturalists. The same spirit finds its fullest expression in English institutions of the same and the following period: the Royal Geographic Society with its celebration of David Livinsgtone; the Kew Gardens; the Natural History Museum; and even the British Museum, founded by Sir Hans Sloane, an Irish physician and botanist. The latter figure is particularly interesting: the first collections housed in the embryonic Natural History Museum in London were in fact assembled by him and donated to the British government. The collection included dried plants and skeletons of animals and humans and was first displayed in 1756. But Sloane was also the founder of the British Museum, and he is largely responsible for what is now the Enlightenment Gallery collection. The Gallery is unique in that it brings together objects from all the then known world, from Japan to Chile, mixing artifacts with objects of naturalistic interest. It is the ultimate expression of the age of colonialism, with all its connotations: universalism (with the British Empire at the center), extractive inclination, genuine aesthetic curiosity, classificatory and scientific interest.

Health was not extraneous to these huge historical changes. In the era of great navigations it was harbors that were the crossroads of exchanges and intermingling of genetically and culturally diverse populations, goods, commodities, works of art, and microbes. The plague in Venice or the cholera in Hamburg are obvious representations: major epidemics were transmitted by sea, and they were associated with trade conflicts. Indeed, the opening of the Suez Canal by Lesseps led both to an intensification of cholera epidemics in Europe (from the Indies) and to “policing” measures. For example, in the years after 1866 the authorities regulating the flow of ships for sanitary reasons required English ships to stop in order to prevent the spread of the epidemic, but the British refused, arguing that this was an act of commercial warfare. The British behavior contradicted the results of the cholera conferences in Constantinople (1866) and Vienna (1874). Indeed, the British argued that Indian cholera was a noncontagious disease, thus providing a clear example of *bio-geopolitics* (2).

**Figure 1:**
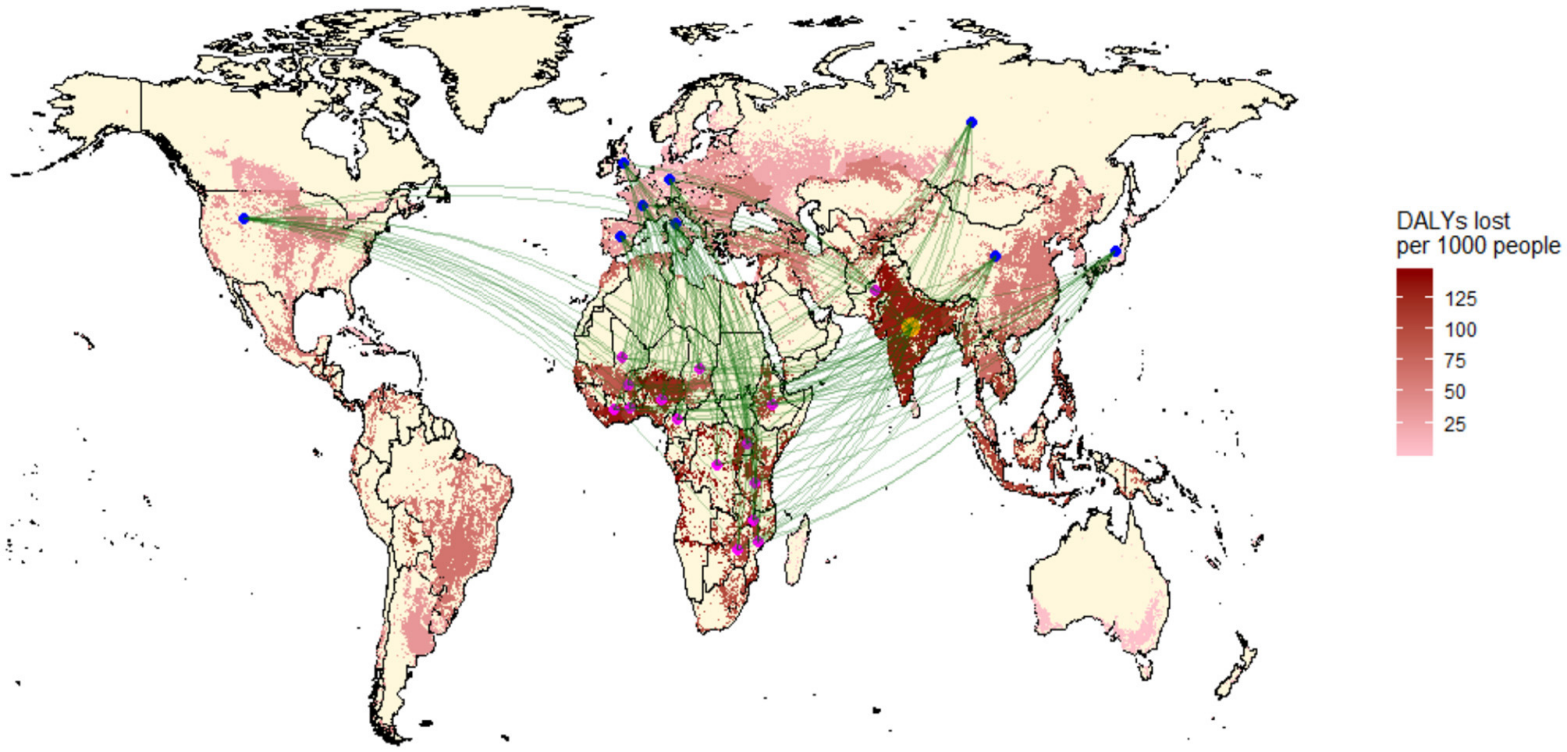
Flow map of the top trade routes of disease implicated commodities. Note that the green lines directly link the agriculture producing countries, where disease burdens are incurred (magenta circles), and the top nine final consumer countries (blue circles). India is both an importer and sufferer (courtesy H Shah, K Murray).

Today, compared to the era of shipping, the scale has completely changed, as Figure 1 shows, representing the impact of international trades (by ship and air) on zoonoses in low-income countries. The world is divided into countries importing goods (in this case mostly foodstuffs, but also raw materials such as rubber) and countries subject to extractive activities, in which zoonoses occur more frequently because of land degradation and increased spillovers.

The problem is that not everything about the biosphere and especially the geosphere is regenerable except over many centuries. The mechanism of progressive human colonization of the earth's surface and sea is at the roots of constant and progressive imbalances that the planet as such cannot repair. Many natural phenomena follow non-linear relationships, that is, there is no proportionality between cause and effect, or even there are “*tipping points*” that accelerate the occurrence and magnitude of effects. In the case of the biosphere, a change in state (after a *tipping point* is reached) might be the melting of permafrost with a rapid release of methane and further warming of the Earth. Or the level beyond which the Amazon rainforest - undergoing rapid deforestation - turns into a savannah. Also this event would be catastrophic because it would correspond to a total change in the habitat of a large number of animal and plant species. In a study published recently, satellite images report how since the early 2000s more than 75% of the Amazon rainforest shows signs of loss of resilience of ecosystems, for example against drought or heat waves (3). When such a progressive depletion will lead to the transformation to savannah is not known. What is possible is that further nonlinear phenomena will be triggered in this case as well, namely the release of billions more tons of CO_2_ and thus an acceleration of the climate crisis.

What about inequalities? In his latest work Piketty reconstructs the trades and economic exchanges between the current low-income countries and the rest of the world, between the eighteenth and the twenty first centuries. To exemplify the transition that occurred, China and India in 1800 shared 53% of world trades, that became only 5% in 1900 (1). The seminal work by Kenneth Pomeranz (4) shows that around 1830 more than 10 million hectares of arable land in the American plantations were used for the export of wood, cotton and sugar to England, corresponding to between 1 ½ and 2 times the arable land of the latter. In fact, the Western industrialization was made possible by a mobilization of resources and workforce on a planetary scale. Piketty's conclusion is that we have just started emerging from this great “colonial experiment”.

Many consequences of the colonial and post-colonial economic model described above are demonstrated by the “Great Acceleration” of human-driven changes to the Earth System, also marking the start of what has been termed the “Anthropocene” (5). Specifically, the growth in a number of socio-economic indicators, such as GDP, population, primary energy use, water use, fertilizer use, transport and communications, has accelerated since 1950, along with environmental impacts including temperature change, ocean acidification and terrestrial biosphere degradation. The overarching socio-economic indicator “growth” also masks huge inequalities, with most of the population growth during this period occurring in the non-OECD world, whilst most of the GDP growth has been in the OECD.

## Our planetary evolutionary niche

The theory of the human “planetary evolutionary niche” has got traction in recent years (6). According to the theory, unlike all other species we have extended our ecological niche to the whole planet. Ecological niches of other species imply a network of co-evolution between microbes, plants and animals, and the system overall tends to be in a dynamic equilibrium, with symbiotic circularity across species. Our planet today – dominated by human action - is characterized by extraction of resources very far away from where they are processed and utilized, and the main form of circularity is transferring back residues of production which are usually of no value for the recipients (like African countries). Over the years, demand has driven the loss of millions of hectares of forests, savannahs and grasslands, particularly in tropical areas, destroying valuable ecosystems and contributing significantly to climate change and biodiversity loss. The European Union is the second-largest importer of agricultural commodities associated with deforestation, after China. Between 2005 and 2017, millions of hectares of forest were lost to produce goods for the EU market, leading to the release of an estimated 1,807 million tons of CO2 (7). Pendrill et al. (8) have computed that about one-sixth of the carbon footprint of the average diet in the European Union can be linked directly to deforestation in tropical countries. From climate change and forest fires to extensive losses in biodiversity, the environmental consequences of deforestation are well documented.

## A new economic theory

Research and policies have tended so far to be practiced in silos. However, historical work shows the intersection of environmental, social, gender and racial inequalities. A deeper look also shows the inadequacy of traditional, liberal economic theory. There are several versions of alternative economic theories, but here we refer to one in particular, put forward by the Italian economist Stefano Zamagni (9). According to this version, the mainstream economic tradition has been built upon the principle of the *exchange of equivalents* (essentially money) taking the place previously occupied by the *principle of reciprocity*, the pillar of the pre-modern social order for Zamagni. The anonymous and impersonal market would have replaced the “person-centered pre-modern communities”. This school of economics proposes a “Civil Economy” where civil comes from the Latin civitas, that is the translation of the Greek polis. According to Zamagni, civil virtues are, since Aristotle, the preconditions for happiness, but from Bentham onwards the classic concept of happiness as “*eudaimonia*” has been reduced to “*utility*”. To define economics as the “science of utility” has deprived economists of the categories of thought for properly tackling social interactions, relationality and reciprocity. Happiness is constitutionally a “relational” entity, while utility is the property of the “relation between a human and a thing”.

Whether or not we agree with this reconstruction of the history of economics, the same considerations are transferable to research and policies dealing with the environmental crisis. According to the Dasgupta review on “The economics of biodiversity” (10), “Inclusive wealth is the measure of an economy's productive capacity. If the inclusive wealth per capita we bequeath to our descendants is greater than the inclusive wealth per capita we ourselves inherited, we would be leaving behind a larger productive base for each of our Descendants. Being an aggregate figure, inclusive wealth does not reflect its distribution across people. An enormous literature on distributive justice can be brought to bear to sharpen sustainability assessment and policy analysis. In doing that though, the citizen investor would be reading the distribution of inclusive wealth, not the distribution of GDP” (10). It is clear that inclusive wealth is a relational concept, not simply the relationship between humans and things.

## An alliance between policy-makers, youth movements, business world, scientists

If we believe, based on the historical considerations above, that poverty, inequalities and the environmental crisis have the same roots, we need a new political economy inspired by the idea of “Civil Economics”, that is an economic theory that goes beyond the liberal tradition. Rather than being “extractive” it is supposed to be inspired by circularity, i.e., replacement of the resources that have been depleted. This requires not only a revolution in production, but also a system of indicators that supports the change and allows policy-makers and society at large to monitor progress (a tendency in this direction is represented by the new European CSRD - Corporate Sustainability Reporting Directive). The new economics would also require cooperation by scientists in providing effective solutions that allow resource regeneration in the productive system. It should look at human-to-human relationships rather than human-to-objects relationships, where humans include future generations. This is a key point. The idea of immediate utility and the current consumeristic approach (“humans-to-things” relationships) show considerable difficulties in founding the idea of responsibility toward future generations.

In fact, we have now a number of promising roots from which to build this new economics. For example, we have had ecological economics since the 1970s (11), and now we face a growing appreciation and re-validation of such concepts, via alternative economic paradigms such as “Doughnut Economics” (12), and “post-growth” (13). The conceptual roots of more planetary-friendly and equitable economics are now being taken increasingly seriously, for example in the European Union through the Green Deal (14).

However, until we do not address the ethical dilemma of responsibilities toward both present and future generations a serious alliance between those who control economic power, productive means and political levers with the youth movements will be impossible or hypocritical. This requires – *inter alia*- much greater investment in education, to create the common language for such an alliance, including transfer to younger generations of key knowledge on the sustainable development goals (SDGs) as encompassed in Agenda 2030 and the Green Deal (15).

## Author contributions

All authors listed have made a substantial, direct, and intellectual contribution to the work and approved it for publication.
